# Feasibility of left bundle branch area pacing with left axillary pacemaker implantation in a young female patient with heart failure

**DOI:** 10.1002/joa3.70022

**Published:** 2025-02-07

**Authors:** Hiroyuki Kato, Yuji Narita, Satoshi Yanagisawa, Yasuya Inden, Toyoaki Murohara

**Affiliations:** ^1^ Department of Cardiology Japan Community Health Care Organization Chukyo Hospital Nagoya Japan; ^2^ Department of Cardiac Surgery Nagoya University Graduate School of Medicine Nagoya Japan; ^3^ Department of Cardiology Nagoya University Graduate School of Medicine Nagoya Japan

**Keywords:** atrioventricular block, axillary generator, conduction system pacing, cosmetic approach, two‐incision approach

## Abstract

Device implantation with a generator pocket raises cosmetic concerns regarding external appearance. We present a case of successful left bundle branch area pacing and left axillary pacemaker generator implantation via a two‐incision approach in a young female patient, resulting in favorable cardiac function and cosmetic satisfaction.

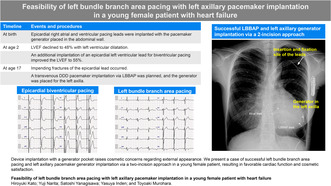

Device implantation with a generator pocket raises cosmetic concerns with respect to external appearance.^1^ Various device implantation techniques via a cosmetic approach, involving left axillary generator implantation, have been investigated over the years to reduce the patient's cosmetic concerns.^1^ Meanwhile, left bundle branch area pacing (LBBAP), as a form of physiologic pacing, has rapidly developed in recent years for heart failure prevention.^2^, ^3^ However, the feasibility of combining LBBAP lead placement, which requires the use of a delivery sheath, and left axillary generator implantation is unclear. Here, we present a case of successful LBBAP and left axillary pacemaker generator implantation via a two‐incision approach in a young female patient, resulting in favorable cardiac function and cosmetic outcomes.

A 17‐year‐old girl with a congenital atrioventricular block who had previously received epicardial biventricular pacing (BVP) was admitted for a switch to a transvenous pacemaker system due to epicardial lead impending fractures. At birth, she had undergone epicardial right ventricular (RV) pacing with the pacemaker generator placed in the abdominal wall. By the age of 2 years, her left ventricular ejection fraction (LVEF) had declined to 48%, prompting the addition of an epicardial left ventricular lead for BVP. This intervention improved her LVEF to 55% (Figure 1A and Video S1). Prior to the transvenous pacemaker implantation, the healthcare team, the patient, and her parents carefully discussed treatment options. They obtained informed consent to implant a DDD pacemaker via LBBAP to minimize the risk of pacing‐induced cardiomyopathy (PICM).^3^ To improve cosmetic outcomes for the young patient, the generator was planned for implantation in the left axilla.

**FIGURE 1:**
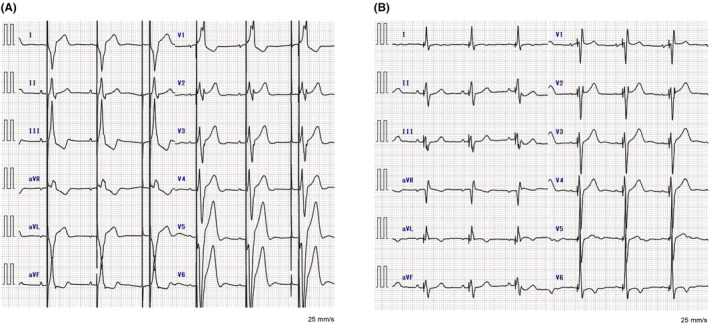
Twelve‐lead electrocardiogram. (A) Preoperative electrocardiogram during biventricular pacing via epicardial electrodes, showing a QRS duration of 188 ms. Despite signs of an impending lead fracture indicated by an increase in lead impedance, biventricular pacing was maintained as there were no failures in sensing or pacing. (B) Postoperative electrocardiogram during left bundle branch area pacing using a bipolar configuration. The QRS duration was significantly reduced to 125 ms.

A two‐incision approach under deep sedation was employed.^4^ Initially, a 1.5 cm subcutaneous pocket was created in the left infraclavicular region. Two guidewires were introduced into the left subclavian vein by puncturing it within the pocket area using the extrathoracic puncture technique. An LBBAP lead was positioned using a lumenless lead (model 3830–69 cm; Medtronic, Minneapolis, MN, USA) and a specialized sheath (model C315HIS; Medtronic). After several attempts, the LBBAP lead was successfully implanted with left bundle branch (LBB) capture (Figure 2A).^3^ An atrial lead (model 3830–69 cm, Medtronic) was implanted in the atrial septum using the C315HIS sheath (Medtronic). Both leads were secured with an anchoring sleeve in the infraclavicular pocket. Next, while elevating the patient's left arm horizontally, a 4.0 cm transverse incision was made, centered on the midaxillary line and aligned with the axillary folds. A left axillary pocket for generator placement was then created beneath the fascia. The atrial and LBBAP leads were routed to this pocket through a subcutaneous tunnel. After connecting the generator (Azure XT DR, Medtronic) to the two leads, it was placed in the axillary pocket, and both incisions were closed. The device was programmed to DDD mode with a rate limit of 50–150 bpm. Sensed and paced atrioventricular intervals were set at 120 and 150 ms, respectively.

**FIGURE 2:**
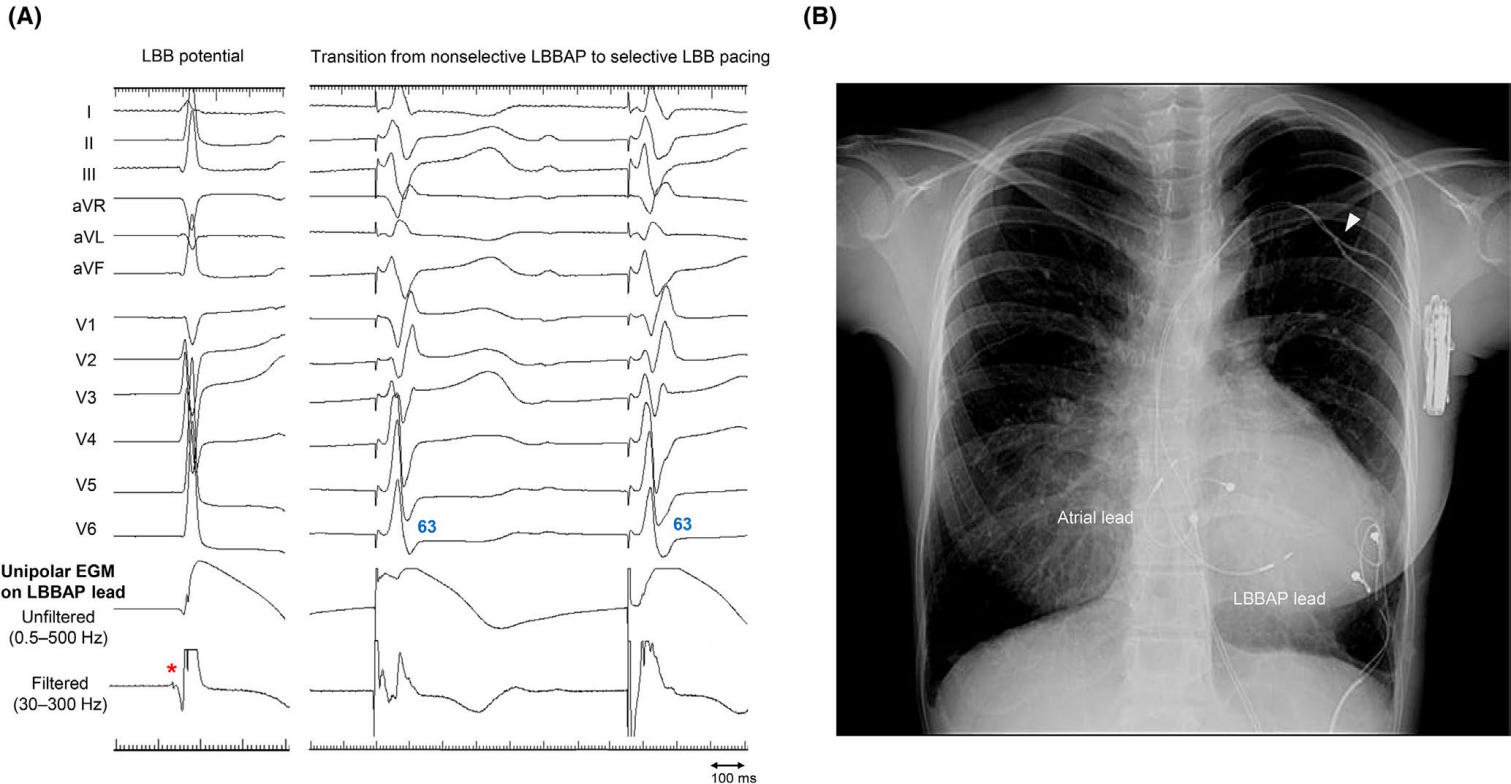
Intraoperative findings and postoperative chest radiography. (A) Left bundle branch (LBB) potential (*red asterisk*) followed by a junctional escape beat after deep lead deployment. The unipolar impedance was 460 Ω, and the amplitude of myocardial injury current was 13.8 mV. During threshold testing at 1.5 V, pacing showed the transition from non‐selective left bundle branch area pacing (LBBAP) to selective LBB pacing, with a constant R‐wave peak time in lead V6 of 63 ms and a split electrogram (EGM). (B) Chest radiography after left bundle branch area lead implantation. The *white arrowhead* marks the infraclavicular pocket site.

The chest radiography image obtained after LBBAP lead implantation is displayed in Figure 2B. Remarkably, the LBB‐paced QRS interval was shorter than that of the preoperative epicardial BVP interval (Figure 1B). No perioperative complications, including lead dislodgement, hematoma, and infection, occurred. At 3 months postoperatively, she underwent an echocardiography, which demonstrated that the LVEF improved to 63% (Video S1). Lead parameters remained stable, with an LBB capture threshold of 0.5 V at 0.4 ms and a ventricular amplitude of 10.4 mV. The patient expressed high satisfaction with the cosmetic outcome, as the pacemaker generator remained concealed in the left axilla (Figure 3).

**FIGURE 3:**
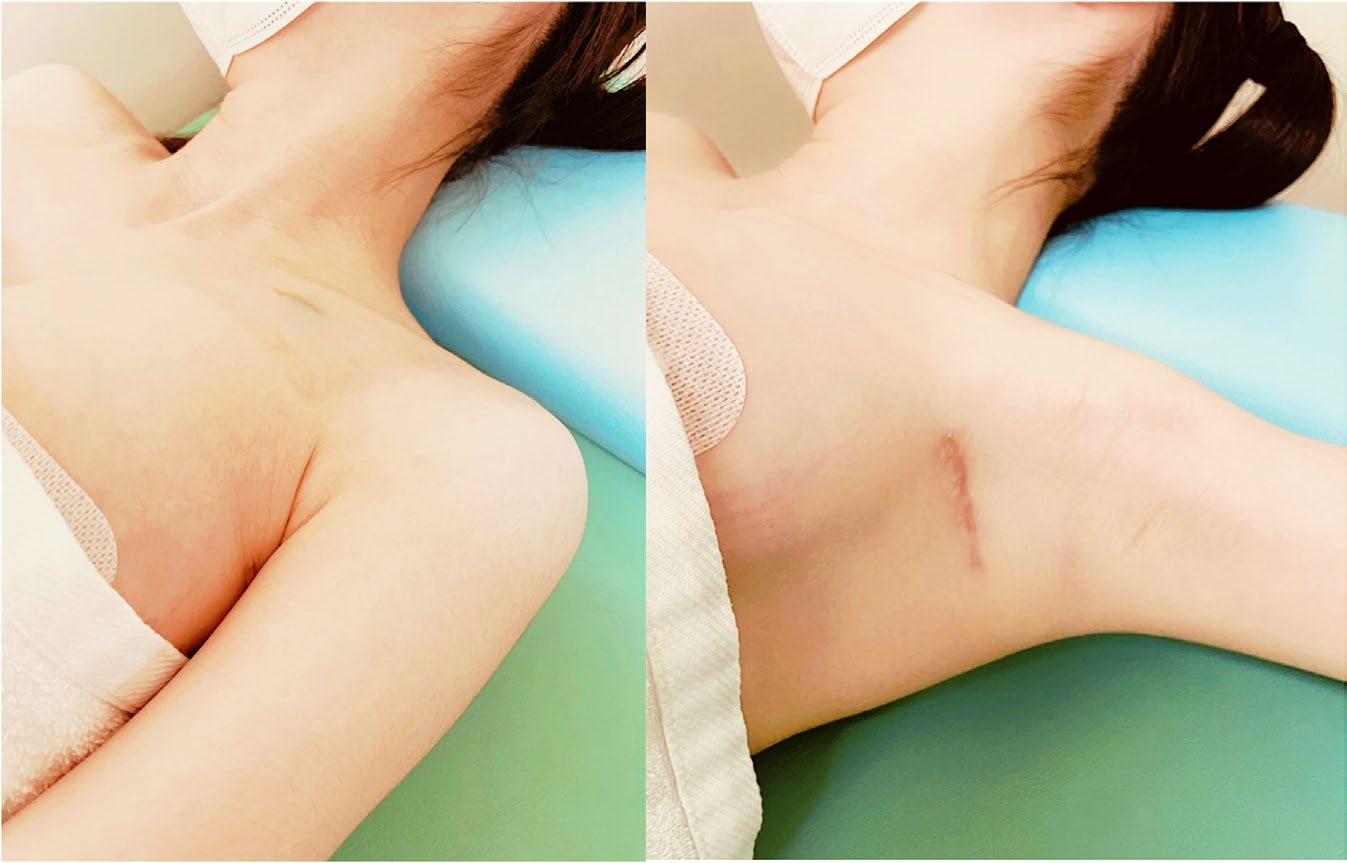
Photographs of the implantation site 3 months post‐surgery. The infraclavicular scar from the LBBAP lead placement is approximately 1.5 cm. The axillary incision scar for the generator is visible only when the patient raises her arm, and the generator's silhouette is not discernible.

Pacemaker generator implantation in the conventional infraclavicular area often poses cosmetic concerns, impacting body image and potentially compelling patients to alter their lifestyles. Therefore, axillary generator implantation can offer distinct advantages.

A one‐incision approach with simultaneous axillary vein puncture may offer superior cosmetic results compared to the two‐incision method.^1^, ^5^ From a different perspective, the two‐incision approach carries a potential heightened risk of lead damage due to the length of the implanted leads in the subcutaneous tunnel.^1^ Nevertheless, in this case, the two‐incision method was selected because using a delivery sheath for LBBAP lead implantation via the axillary vein was deemed unsuitable due to potential issues with sheath length and maneuverability. Additionally, it is pertinent to consider future lead extraction and reimplantation procedures, given the unlikely event that the leads will function for the entire lifespan of this adolescent patient. Anticipated concerns with the one‐incision approach during lead extraction include lead adhesion in the axillary vein, an increased number of bends to the ventricle, and the insufficient length of the device extraction tool.

In the present case, we first considered implanting transvenous RV and coronary sinus leads for BVP. However, we ultimately decided on LBBAP using a DDD pacemaker. A significant advantage of LBBAP over BVP is that LBBAP requires only one ventricular lead. Few implanted pacing leads may reduce the risk of device infection and future venous occlusion. In addition, battery longevity is a crucial consideration, especially for young patients. Left bundle branch area pacing, characterized by its low capture threshold, is expected to enhance battery longevity compared to conventional BVP, which requires pacing from both. From these viewpoints, we believe that selecting LBBAP offers substantial benefits to the patient.

This is the first documented case demonstrating the feasibility of combining LBBAP lead placement with left axillary generator implantation. This novel combination provided the patient with cosmetic satisfaction and positively impacted cardiac function. We believe that this combined approach may be applied to all individuals who require it, regardless of age or gender. Meanwhile, the long‐term safety, durability, and clinical outcomes of the LBBAP lead with a left axillary generator are unclear, awaiting longer follow‐up and further cumulative experience.

## FUNDING INFORMATION

The authors did not receive support from any organization for the submitted work.

## CONFLICT OF INTEREST STATEMENT

Dr. Yanagisawa is affiliated with a department‐sponsored by Medtronic, Japan. Other authors have no conflicts of interest.

## ETHICS APPROVAL STATEMENT

This study was approved by the ethics committee of Chukyo Hospital. The study was performed in accordance with the principles of the Declaration of Helsinki.

## PATIENT CONSENT STATEMENT

The authors confirm that written consent for submission and publication of this case report, including images and associated text, has been obtained from the patient.

## Supporting information


Video S1

## Data Availability

The datasets generated and/or analyzed during the current study are available from the corresponding author on reasonable request.
