# Supplementary material for: Feasibility of left bundle branch area pacing with left axillary pacemaker implantation in a young female patient with heart failure
Source: J Arrhythm. 2025 Feb 7;41(1):e70022. doi: 10.1002/joa3.70022 (PMC11803607; doi:10.1002/joa3.70022)
Supplement: Supplementary file 1 — Video S1 [file JOA3-41-e70022-s001.zip › Video_legend.docx]

**Video S1: Echocardiography during biventricular pacing via epicardial electrodes and after left bundle branch area pacing.** Echocardiography on a parasternal short-axis view during epicardial BVP. The LVEF improved to 55% after BVP, following decreased LVEF of 48% preoperatively. Echocardiography on a parasternal short-axis view 3 months after LBBAP revealed that the LVEF further improved to 63%. Note that the LBBAP lead tip is positioned near the left ventricular endocardium. BVP, biventricular pacing; LBBAP, left bundle branch area pacing; LVEF, left ventricular ejection fraction.
